# Synthesis of Colloidal Au Nanoparticles through Ultrasonic Spray Pyrolysis and Their Use in the Preparation of Polyacrylate-AuNPs’ Composites

**DOI:** 10.3390/ma12223775

**Published:** 2019-11-17

**Authors:** Doris Golub, Andrej Ivanič, Peter Majerič, Hanuma Reddy Tiyyagura, Ivan Anžel, Rebeka Rudolf

**Affiliations:** 1 Zlatarna Celje d. o. o., Kersnikova 19, 3000 Celje, Slovenia; hanuma.tiyyagura@um.si (H.R.T.); rebeka.rudolf@um.si (R.R.); 2 Faculty of Civil Engineering, Transportation Engineering and Architecture, University of Maribor, Smetanova ulica 17, 2000 Maribor, Slovenia; andrej.ivanic@um.si; 3 Faculty of Mechanical Engineering, University of Maribor, Smetanova ulica 17, 2000 Maribor, Slovenia; peter.majeric@um.si (P.M.); ivan.anzel@um.si (I.A.)

**Keywords:** Ultrasonic Spray Pyrolysis, colloidal gold nanoparticles, polyacrylate composites, characterization

## Abstract

Colloidal gold nanoparticles (AuNPs) were prepared from two different liquid precursors (gold (III) acetate and gold (III) chloride), using the Ultrasonic Spray Pyrolysis (USP) process. The STEM characterisation showed that the AuNPs from gold chloride are spherical, with average diameters of 57.2 and 69.4 nm, while the AuNPs from gold acetate are ellipsoidal, with average diameters of 84.2 and 134.3 nm, according to Dynamic Light Scattering (DLS) measurements. UV/VIS spectroscopy revealed the maximum absorbance band of AuNPs between 532 and 560 nm, which indicates a stable state. Colloidal AuNPs were used as starting material and were mixed together with acrylic acid (AA) and acrylamide (Am) for the free radical polymerization of polyacrylate-AuNPs’ composites, with the purpose of using them for temporary cavity fillings in the dental industry. SEM characterisation of polyacrylate-AuNPs’ composites revealed a uniform distribution of AuNPs through the polymer matrix, revealing that the AuNPs remained stable during the polymerization process. The density measurements revealed that colloidal AuNPs increase the densities of the prepared polyacrylate-AuNPs’ composites; the densities were increased up to 40% in comparison with the densities of the control samples. A compressive test showed that polyacrylate-AuNPs’ composites exhibited lower compressive strength compared to the control samples, while their toughness increased. At 50% compression deformation some of the samples fracture, suggesting that incorporation of colloidal AuNPs do not improve their compressive strength, but increase their toughness significantly. This increased toughness is the measured property which makes prepared polyacrylate-AuNPs potentially useful in dentistry.

## 1. Introduction

Over the past two decades, metal nanoparticles, especially gold nanoparticles (AuNPs), have attracted a lot of attention from the scientific community due to their specific and unique chemical, physical, photochemical, optical and electronic properties, which differ from the properties of materials in their bulk state [1]. AuNPs’ unique properties include high surface area to volume ratio, high chemical and physical stability, high charge density, and a phenomenon known as Surface Plasmon Resonance (SPR) [2,3,4,5,6,7]. AuNPs are also biocompatible, biologically unreactive, and can be functionalised with medical drugs, enzymes and proteins [8,9]. Because of this, they can be used in various biomedical fields, such as drug and gene delivery [10,11,12,13], tissue imaging [8], therapeutic agents [14,15], diagnosis treatment [16], sensors and biosensors [17,18], catalysts [19,20,21,22,23] and in dentistry [16,24,25,26,27]. 

The physicochemical properties of AuNPs are determined by their morphology, size, purity and surface structure [28], and in recent years a number of methods have been developed for the synthesis of AuNPs. These methods are commonly divided into bottom-up and top-down approaches [4,7,9,29,30,31]. In the bottom-up approach AuNPs are formed from molecular (atomic) components (metal ions), and the most typical bottom-up approaches are chemical vapour deposition [32], atomic layer deposition [33], flame spray synthesis [34,35], the sol-gel process [36] and various pyrolysis processes [37,38,39]. Meanwhile, in the top-down approach, the AuNPs are produced from the starting (bulk) materials. The most common methods for the top-down approach are mechanical grinding and ball-milling, chemical etching, nanolithography and laser ablation [28,29,40,41]. All of these methods are suitable for the production of small quantities of nanoparticles, whose sizes and shapes are dependent on the used batch. 

One of the bottom-up methods that has a great potential for mass production of AuNPs from various materials is Ultrasonic Spray Pyrolysis (USP). USP is a relatively simple and flexible technique for the production of nanoparticles, and the properties of nanoparticles can be controlled by modification of the USP process parameters [4,28,29,37,38,39,42]. USP is also more economical than other processes, as it operates at ambient pressure and temperature [39,42]. With USP, nanoparticles are synthesised from droplets of metal salt precursor solution, generated with ultrasound. These aerosol droplets are then transported by carrier gas (nitrogen) into the heating chambers following their chemical decomposition at elevated temperatures [24] and their collection in a suitable collection medium [43]. The collection mediums usually contain stabilising agents such as sodium citrate, polyvinylpyrrolidone (PVP) or polyethylene glycol (PEG), which prevent further reactions of nanoparticles (like oxidation) as they stabilise nanoparticles and prevent their agglomeration [28].

The most commonly used metal salt precursor for production of AuNPs is tetrachloro-auric (III) acid in hydrate form (also known as gold (III) chloride or HAuCl_4_ × nH_2_O) due to its solubility in water, although many other chloride-free precursors can be used, such as gold (III) acetate (Au(CH_3_COO)_3_), gold (I) bromide (AuBr), gold (III) oxide and gold (III) nitrate (Au(NO_3_)_3_), as already reported [39,44,45]. These gold salts are solid and crystalline, partially insoluble in common organic solvents, and they usually decompose in water [39]. 

Over the past few years, nanotechnology, and, thus, the use of nanomaterials, has become an invaluable part of dentistry. Nanomaterials in dentistry can help with diagnosing, treating and preventing dental and oral diseases [46]. Among the most used materials in dentistry are polymeric materials, which can be tailored for a wide range of applications, due to their mechanical and biological properties, affordability and ease in processing [47]. An extensively used polymeric material in dentistry is polymethyl methacrylate (PMMA), which is a strong and tough material, is easy to use and is inexpensive [47,48,49,50]. Other polymeric material types for dental applications are poly(acrylic) acid (PAA) and polyacrylamide (PAm), usually prepared by the free radical polymerization of the monomer (acrylic acid or acrylamide) in aqueous solution [51,52,53]. These materials are used mainly in the fields of Prosthetics, Orthodontics and Periodontics, in the form of nanocomposites, nano-ceramics, dental adhesives and cements, fillers, dentures and implants [52]. 

Inorganic (metal) nanoparticles can act as antimicrobial agents, since they have the ability to interact with microorganisms, and their antimicrobial activity can be attributed to the cytotoxicity of various bacterial cells [54,55]. In addition to their biocompatibility, AuNPs are also non-toxic and anti-inflammatory; studies showed that AuNPs exhibit antifungal and antibacterial activity against various fungi and bacteria that causes different oral diseases [55,56,57,58], while this activity appeared to be size, shape and concentration dependent. Thus, AuNPs are great candidates for dental applications. The incorporation of AuNPs into dental composites can inhibit bacterial growth, and also improve their physical properties, like mechanical strength, toughness and durability [47,59].

The aim of this study was the preparation of colloidal AuNPs from two different liquid precursors (gold (III) chloride and gold (III) acetate), using a modified USP process, for their use in the preparation of polyacrylate-AuNPs composites, to be used as potential material for temporary cavity fillings in the dental industry. The physical properties of the prepared colloidal AuNPs, and the morphology, density and mechanical properties of the prepared polyacrylate-AuNPs’ composites, were investigated using different characterisation techniques (Scanning Electron Microscopy (SEM), Scanning Transmission Electron Microscopy (STEM), Transmission Electron Microscopy (TEM), Dynamic Light Scattering (DLS), ultraviolet/visible (UV/VIS) spectroscopy, density measurements and mechanical testing). 

## 2. Materials and Methods

### 2.1. Precursor Solutions’ Preparation and USP Synthesis

Precursor solutions of gold were prepared from two different gold salts, tetrachloro-auric (III) acid trihydrate (HAuCl_4_ × 3 H_2_O or gold (III) chloride (AuCl), Acros Organics, Geel, Belgium) and gold (III) acetate ((Au(CH_3_COO)_3_) or AuAc, Alfa Aesar, Kandel, Germany). The procedure for preparation of the chloride precursor solution was as follows: HAuCl_4_ salt was dissolved in deionised water by stirring with a magnetic stirrer, until a clear yellow solution was obtained. The concentration of Au in the chloride precursor solution was 1 g/L. The acetate precursor solution was prepared by dissolving AuAc in deionised water mixed together with hydrochloric acid (HCl, 37%, Sigma Aldrich, Taufkirchen, Germany), and stirring magnetically until a clear yellow solution was obtained. The obtained precursor solution was too acidic for direct use in the USP system (pH value is in the range of 1–2), therefore, sodium hydroxide (NaOH, Fisher Chemicals, Geel, Belgium) in the form of pellets was used to increase the pH value to the desired range of 5–6. The concentration of Au in the acetate precursor solution was 1 g/L. 

**Figure 1:**
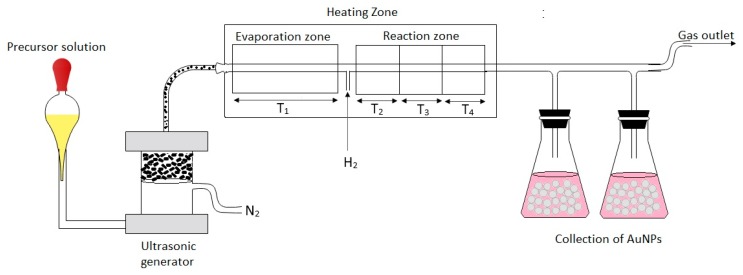
Modified Ultrasonic Spray Pyrolysis (USP) for AuNPs’ synthesis.

The synthesis of colloidal gold nanoparticles (AuNPs) was carried out on the modified USP equipment at Zlatarna Celje d.o.o., Slovenia (Figure 1). This modified USP consists of an ultrasonic nebulizer, heating zone (evaporation zone and reaction zone), collecting bottles and transport tubes. The USP furnace was modified to separate the heating zone into four zones, separating the evaporation of aerosol droplets in the evaporation zone from the particle drying in the reaction zones. The temperatures were maintained at T_1_, T_2_, T_3_ and T_4_, with T_1_ being the temperature in the evaporation zone, while the remaining temperatures represented the temperatures in the reaction zone. The prepared precursor solutions were poured into the ultrasonic nebulizer with a frequency of 2.5 MHz, where the aerosol droplets were generated. The aerosol droplets were then transported through a quartz tube to the heating zones using nitrogen (N_2_) as the carrier gas, while hydrogen (H_2_) was used for the reduction into pure AuNPs. T_1_ was kept at lower temperatures, while T_2_, T_3_ and T_4_ were set at the higher temperatures required for nanoparticle synthesis. Table 1 shows all the parameters used in the USP synthesis. 

The synthesised AuNPs were collected in collection bottles, where 0.1% solution of polyvinylpyrrolidone (PVP) in deionised water was used as the collecting medium, to prevent AuNPs’ agglomeration.

### 2.2. Preparation of Polyacrylate-AuNPs’ Composites

For the preparation of polyacrylate-AuNPs’ composites, two different monomers, namely acrylic acid (AA, 98%, Acros Organics, Geel, Belgium) and acrylamide (Am, 99%, Sigma Aldrich, Taufkirchen, Germany), and two different initiators were used, namely ammonium persulfate (APS, 98%, Acros Organics, Geel, Belgium) and potassium persulfate (KPS, 99%, Acros Organics, Geel, Belgium). The AA was neutralised prior to use with NaOH pellets dissolved in 1.5 mL deionised water, to achieve a pH value of around 6. The basic procedure for polyacrylate-AuNPs’ composites’ preparation was as follows: The monomer (neutralised AA or Am) and colloidal AuNPs were mixed together in a round bottom flask at room temperature using a magnetic stirrer at 280 rpm. When the mixture was homogeneous, the initiator (APS or KPS) was added, and the stirring was continued for a further 30 min, to ensure that all the initiator was dissolved. Then one drop of the reducing agent N,N,N′,N′-tetramethylethylenediamine (TEMED, 99.5 %, Acros Organics, Geel, Belgium) was added, and the mixture was transferred quickly into moulds and cured in a water bath at 60 °C for 3 h. After the polymerization was complete, the moulds were cooled to room temperature and the polyacrylate-AuNPs’ composites were removed.

Control samples without AuNPs were also prepared in addition to the polyacrylate-AuNPs’ composites. They were prepared with the same procedure as the polyacrylate-AuNPs’ composites, except that deionised water was used as the solvent instead of colloidal AuNPs. The composition data for control samples and prepared polyacrylate-AuNPs composites are presented in Table 2.

### 2.3. Characterisation of Prepared AuNPs 

The nature of the synthesised AuNPs, such as their size, shape and morphology is significant in the evaluation and characterisation of the prepared AuNPs [60]. Firstly, the morphology of synthesised colloidal AuNPs was studied by SEM Sirion 400NC (FEI, Hillsboro, OR, USA) using a STEM detector. A drop of colloidal suspension of AuNPs in deionised water, stabilised with PVP, was pipetted onto 300 mesh copper TEM grids with a holey carbon film. The grids were then air dried at room temperature for STEM investigations. An electron accelerating voltage of 15 to 30 kV with various beam spot sizes was used, depending on the sample. The hydrodynamic size distributions (using DLS) were investigated with a Malvern Zetasizer Nano ZS apparatus (Malvern Panalytical, Malvern Worcestershire, UK), using single-use plastic cuvettes. The initial parameters for the material were: Refractive Index (R.I.) = 0.2, absorbance = 3.32, while dispersant properties were: Dispersant = water, temperature = 25 °C, R.I. = 1.33, viscosity = 0.88 cP, dielectric constant = 78.5.

One of the main key techniques for evaluation of optical and structural properties of AuNPs is UV/VIS absorption spectroscopy [44], with which the interactions between AuNPs with different electromagnetic waves can be investigated. It is known that the AuNPs have unique optical properties. At a specific wavelength, the electrons on the AuNPs’ surface can fluctuate and cause SPR, which is strongly dependent on the AuNPs’ size, shape and their agglomeration state [61]. Therefore, the UV/VIS absorption of colloidal AuNPs solutions was measured, using a quartz cuvette with a Tecan Infinite M200 UV/VIS spectrophotometer (Tecan, Grödig, Austria). The absorbance measurements were made over the wavelength range of 300–700 nm, with number of flashes = 5× and time per measure = 20 ms. 

### 2.4. Characterisation of Polyacrylate-AuNPs’ Composites

The characterisation and the distribution of AuNPs in the prepared polyacrylate-AuNPs’ composites was performed by examining the fracture surfaces of the polyacrylate-AuNPs’ composites, formed after mechanical tests, ensuring that at least 50% of the AuNPs were retained in the solid matrix of the polyacrylate-AuNPs’ composite. The surface characterisation of polyacrylate- AuNPs’ composites itself does not give a realistic picture of AuNPs’ distribution in the polymer matrix, since, with the surface preparation (grinding and polishing), most of the AuNPs would be removed from the composite surface. The polyacrylate-AuNPs’ composites were put on SEM holders with conductive carbon adhesive tape for observations, and the characterisation was performed using a SEM Sirion 400NC (FEI, Hillsboro, OR, USA) with Energy-Dispersive X-ray (EDX) spectroscope INCA 350 (Oxford Instruments, Abingdon, UK) under the initial parameters: High Vacuum (HV): 15 kV, detector BSE, magnification: 12,500–50,000×. 

The densities of the prepared cylindrical polyacrylate-AuNPs’ composites, with a diameter of 8 mm and length of 10 mm, were measured with a 50 ml Hubbard pycnometer at 20 °C. For polyacrylate-AuNPs’ composites based on AA, pure AA was chosen as the medium for density measurements, while for polyacrylate-AuNPs’ composites based on Am, the chosen medium for density measurements was toluene. Both mediums were chosen because of their inactivity to the samples. The densities of the polyacrylate-AuNPs’ composites were calculated using Equation (1):

ρ_com_ = (m_com_ × ρ_sol_)/(m_(p+sol)_ + (m_com_ − m_(p+sol+com)_) (1)

where ρ_com_ is the density of the composite (g/cm^3^), m_com_ is the mass of the composite (g), ρ_sol_ is the density of the solvent (g/cm^3^), m_(p+sol)_ is the mass of the pycnometer with solvent (g) and m_(p+sol+com)_ is the mass of the pycnometer, solvent and sample (g).

The mechanical properties of the prepared polyacrylate-AuNPs’ composites were performed according to Standard SIST EN ISO 604 [62], and were tested with compressive tests on a ZWICK/ROELL Z010 materials testing machine (Zwick/Roell, Ulm, Germany), at a crosshead rate of 2.0 mm/min. The cylindrically shaped test specimens, with a diameter of 10 mm and 10 mm in length, were compressed along its major axis at a constant speed until the decrease in length reached a predetermined value. The applied load and compressive strain were recorded. The loading of the specimen was stopped at 50% compressive strain, mainly for security reasons. The load-strain responses were used to calculate the compressive strength σ_M_ and toughness. 

## 3. Results and Discussion

### 3.1. Precursor Solutions’ Preparation and USP Synthesis

USP synthesis of colloidal AuNPs from two different gold salts, AuCl and AuAc, was carried out according to the parameters presented in Table 1. Colloidal AuNPs were synthesised under different conditions (reaction zone temperature and gas flow) to analyse the effect of these parameters on the AuNPs’ morphology. In the first experiment, lower temperature and higher gas flow were used, resulting in the formation of smaller AuNPs, which was evident from the colour change of the collecting medium (from transparent to light pink or purple). In the second experiment, the temperature of the reaction zone was increased, while the gas flow was reduced. This ensured an increase in the kinetics of the entire USP process, which resulted in bigger and more stable AuNPs. In the USP process, the ultrasonic generator then formed the aerosol cloud of the precursor solution, also known as primary droplets, which were carried with the carrier gas nitrogen (N_2_) into the evaporation zone, where droplets began to evaporate and shrink, increasing the Au concentration within the evaporated droplets [39,63]. A micro-explosion of primary droplets occurred in the reaction zone, resulting in secondary droplets, which underwent thermal conversion and reduction with the help of hydrogen (H_2_) into pure AuNPs [63]. 

Compared to the AuCl precursor solution, which was prepared simply by the dissolution of the salt in deionised water, the AuAc solution contains acetic acid and chloride acid, along with sodium chloride for raising the pH value of the solution, in order for it to be usable in the ultrasonic generator. Due to these substances in the AuAc precursor solution, the rheological characteristics of the solution (viscosity, density, surface tension), pH and ionic strength are different as compared to the AuCl solution. This, in turn, changes the aerosol droplet size generated by the ultrasound, as well as the droplet evaporation, the formation of nanoparticles from the droplets in the USP process, and their morphology. 

The AuNPs’ formation was confirmed with the colour change of the collecting medium in which the final AuNPs were collected for both experiments.

### 3.2. Characterisation of Prepared AuNPs

#### 3.2.1. STEM and TEM Characterisation

**Figure 2:**
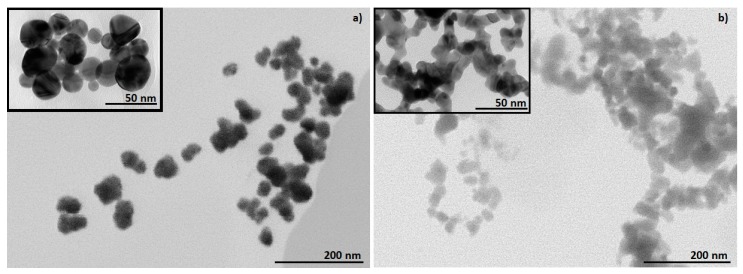
STEM images of colloidal AuNPs, with inserted TEM images: (**a**) AuCl and (**b**) AuAc.

STEM and TEM characterisation provide chemical information of the nanoparticles, revealing the shapes and sizes of the nanomaterials, as well as their dispersion and aggregation/agglomeration degree [64]. STEM and TEM characterisation of colloidal AuNPs confirmed the presence of AuNPs synthesised from precursor solutions with concentrations of [Au] = 1 g/L. From Figure 2 it can be observed that the AuNPs from AuCl are spherical (Figure 2a), while AuNPs from AuAc are ellipsoidal (Figure 2b), although irregularly shaped AuNPs were also observed. When using the AuAc precursor with USP, finer nanoparticles are formed, which appear in mesh-like structures (Figure 2b), most likely due to the collision and sintering of AuNPs in the reaction zone during AuNPs’ synthesis. Meanwhile, the AuNPs from AuCl were more dispersed, where some of the AuNPs were coalesced together in agglomerates by van der Waal forces. There were no visible deformations, like cracks or holes, on the surface of the AuNPs.

#### 3.2.2. Size Distribution by DLS and STEM

**Figure 3:**
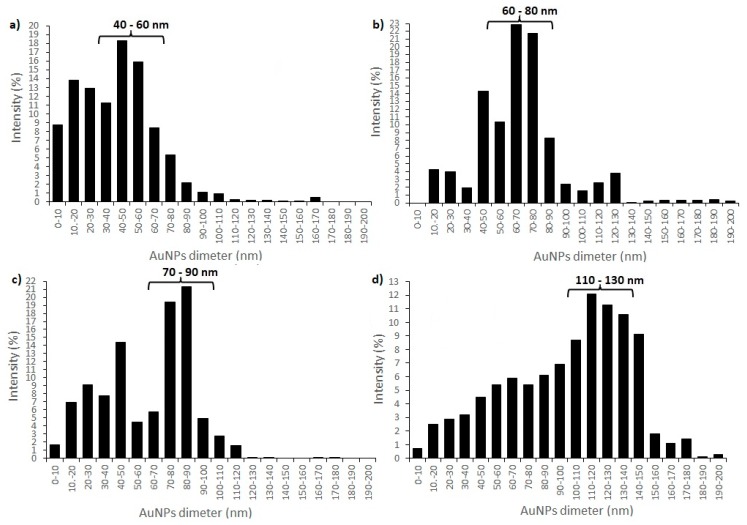
DLS size distributions of colloidal AuNPs: (**a**) AuCl_1; (**b**) AuCl_2; (**c**) AuAc_1 and (**d**) AuAc_2.

With DLS analysis several AuNPs’ characteristics can be revealed, such as shape, structure, hydrodynamic size and agglomeration state, by monitoring the Rayleigh scattering caused by the Brownian motion of AuNPs of a size smaller than the incident light wavelength at a fixed scattering angle [64,65]. DLS analysis of synthesised AuNPs revealed three different groups of AuNPs with different size distributions (in intensity percent) for AuNPs from all precursor solutions. Size distributions for AuNPs from precursor solution AuCl_1 revealed that group 1 contained 8.7% AuNPs with diameter < 10 nm, group 2 contained 56.2% AuNPs with a diameter between 10 and 50 nm, while group 3 contained 35.1% AuNPs with diameter > 50 nm. The average AuNPs’ size (together with Standard Deviation (SD)) of AuCl_1 was 57.2 ± 0.75 nm (Figure 3a). Size distributions for AuNPs from precursor solution AuCl_2 revealed that, in group 1, there were no AuNPs with diameter <10 nm, group 2 contained 24.5% AuNPs with a diameter between 10 and 50 nm, and group 3 contained 75.5% AuNPs with diameter > 50 nm. The average AuNPs’ size (together with Standard Deviation (SD)) of AuCl_2 was 69.4 ± 12.42 nm (Figure 3b). Size distributions for AuNPs from precursor solution AuAc_1 revealed that group 1 contained 1.6% AuNPs with diameter < 10 nm, group 2 contained 38.1% AuNPs with a diameter between 10 and 50 nm, and group 3 contained 60.3% AuNPs with diameter > 50 nm. The average AuNPs’ size (together with Standard Deviation (SD)) of AuAc_1 was 84.2 ± 1.44 nm (Figure 3c). Size distributions for AuNPs from precursor solution AuAc_2 revealed that group 1 contained 0.7% AuNPs with diameter < 10 nm, group 2 contained 13.1% AuNPs with a diameter between 10 and 50 nm, and group 3 contained 86.2% AuNPs with diameter >50 nm. The average AuNPs size (together with Standard Deviation (SD)) of AuAc_2 was 134.3 ± 23.13 nm (Figure 3d). DLS analysis showed that colloidal AuNPs also contained AuNPs whose diameters exceeded 300 nm (below < 1 %), and these sizes were most likely the results of the impurities and stabilizing agent present in AuNPs’ solutions.

The average diameter sizes of AuNPs were also calculated from STEM images by averaging approximately 200 AuNPs and the average mean diameter size (together with Standard Deviation (SD)) of AuNPs were 28.04 ± 5.3 nm for AuCl_1, 33.6 ± 15.1 nm for AuCl_2, 23.1 ± 6.4 nm for AuAc_1 and 46.2 ± 23.2 nm for AuAc_2, respectively. By comparing the average sizes of AuNPs, it can clearly be seen that the average AuNPs sizes from STEM analysis are much lower than the average AuNPs sizes from DLS measurements. This difference is due to the fact that DLS measures hydrodynamic particle size, and the method itself does not distinguish between larger AuNPs and the aggregates of the same size. 

Furthermore, a previous study showed [66] that the pH value of the precursor solutions affects the AuNPs’ sizes, where lower pH values produce smaller AuNPs compared to higher pH values. This was confirmed by the obtained results, since AuNPs from the AuCl precursor solution (pH 2–3) were smaller, compared to AuNPs from the AuAc precursor with a higher pH value (pH 5–6). 

#### 3.2.3. UV/VIS Spectroscopy 

**Figure 4:**
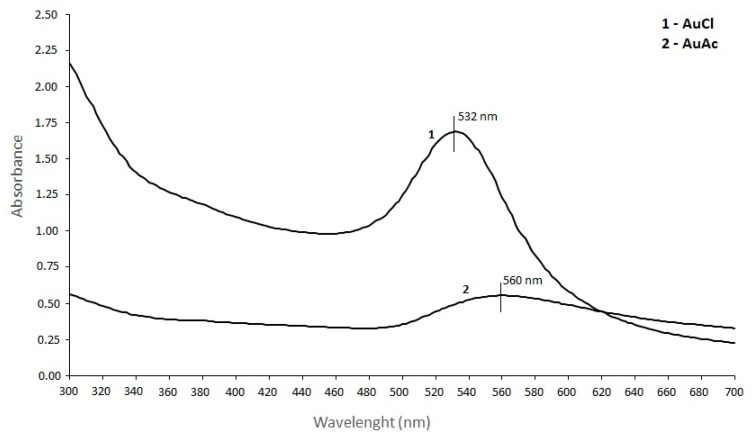
Absorbance spectra of AuNPs from gold chloride and gold acetate.

AuNPs display a single absorption peak in the visible range between 510–570 nm, which gives different coloration due to the AuNPs’ size variations [64]. The UV/VIS spectroscopy of AuNPs was measured between 300–700 nm, and the absorbance spectra can be observed from Figure 4. AuNPs showed the maximum absorption band at ~532 nm for AuNPs from AuCl, and at ~560 nm for AuNPs from AuAc. Both absorption bands indicate the stable state of AuNPs. Also, a shift of the SPR peak to a longer wavelength occurred (from 532 to 560 nm), which is related to the differences in the frequency of SPR oscillations of the free electrons [67]. The increase in absorbance is related to the increase in the nanoparticle diameter. 

### 3.3. Characterisation of Prepared Polyacrylate-AuNPs’ Composites

#### 3.3.1. SEM Characterisation

**Figure 5:**
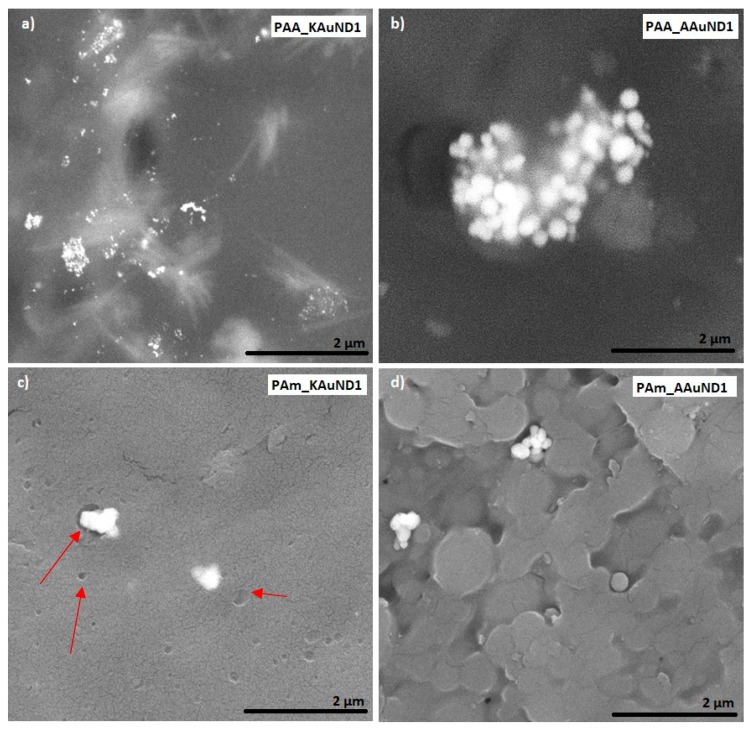
SEM images of prepared polyacrylate-AuNPs’ composites with AuNPs from gold chloride: (**a**) PAA_KAuND1; (**b**) PAA_AAuND1; (**c**) PAm_KAuND1 and (**d**) PAm_AAuND1. The arrows indicate the locations of the microporosity, where AuNPs are located.

**Figure 6:**
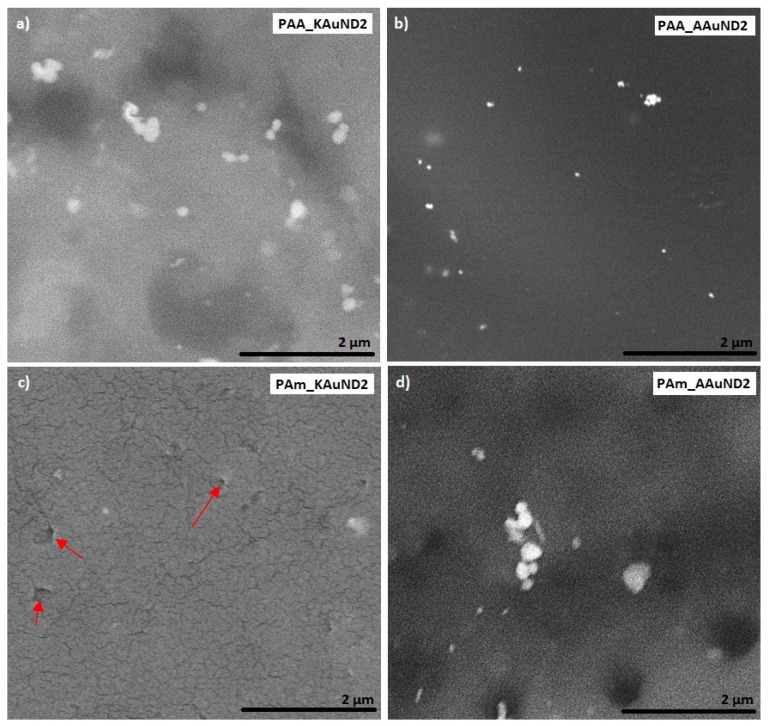
SEM images of prepared polyacrylate-AuNPs’ composites with AuNPs from gold acetate: (**a**) PAA_KAuND2; (**b**) PAA_AAuND2; (**c**) PAm_KAuND2 and (**d**) PAm_AAuND2. The arrows indicate the locations of the microporosity, where AuNPs are located.

SEM characterisation is one of the most used techniques for the examination and analysis of the surface topology and chemical composition of various materials. SEM characterisation was used to investigate the fracture surfaces of polyacrylate-AuNPs’ composites and the distribution of AuNPs in prepared polyacrylate-AuNPs’ composites. The fracture surface morphologies of polyacrylate- AuNPs’ composites prepared with AuNPs from AuCl are presented in Figure 5, and with AuNPs from AuAc in Figure 6. Observations revealed that AuNPs were relatively uniformly distributed (but not ideally) in the composite matrix, with some visible agglomerates and aggregates, indicating that colloidal AuNPs remained mainly stable during the polymerization process. SEM characterisation revealed some small holes and cracks on the polyacrylate-AuNPs’ fracture surfaces, which are the result of generated air bubbles during the radical polymerization process and the mechanical force from the mechanical tests, which caused the fracture.

#### 3.3.2. Density Measurements

The densities of prepared cylindrical polyacrylate-AuNPs’ composites were calculated from Equation (1), and the results are presented in Table 3. As can be seen from the results, AA based polyacrylate-AuNPs’ composites, prepared with APS initiator (PAA_AAuND1 and PAA_AAuND2), have lower densities than AA based polyacrylate-AuNPs’ composites prepared with KPS initiator (PAA_KAuND1 and PAA_KAuND2). On the other hand, Am based polyacrylate-AuNPs’ composites prepared with APS initiator (PAm_AAuND1 and PAm_AAuND2), have lower densities than Am based polyacrylate-AuNPs’ composites prepared with KPS initiator (PAm_KAuND1 and PAm_KAuND2).

The density of AA based composite PAA_KAuND2 (AA, initiator KPS and AuNPs from AuAc) increased by 40.6% compared to the density of the control sample (PAA_KO), while the density of composite PAA_KAuND1 (AA, initiator KPS and AuNPs from AuCl) increased by 17.2% compared to the control sample density. The density of sample PAA_AAuND2 (AA, initiator APS and AuNPs from AuAc) increased by 33.6% compared to the density of the control sample (PAA_AO), while the density of sample PAA_AAuND1 (AA, initiator APS and AuNPs from AuCl) increased by 3.02%.

In the case of polyacrylate-AuNPs’ composites based on Am, results show that the density of composite PAm_KAuND1 (Am, initiator KPS and AuNPs from AuCl) increased by 42.1% compared to the density of the control sample (PAm_KO), while the density of the composite PAm_KAuND2 (Am, initiator KPS and AuNPs from AuAc) increased by 2.58% compared to the density of the control sample. The density of composite PAm_AAuND1 (Am, initiator APS and AuNPs from AuCl) increased by 7.03% compared to the density of the control sample (PAm_AO), while the density of composite PAm_AAuND2 (Am, initiator APS and AuNPs from AuAc) increased by 7.19% compared to the density of the control sample.

Results show that the densities of polyacrylate-AuNPs’ composites increase with the incorporation of colloidal AuNPs. These differences in densities are most likely due to the differences in properties of the chosen colloidal AuNPs, and differences in the initiation mechanisms of the chosen type of initiator (APS or KPS).

#### 3.3.3. Mechanical Properties 

**Figure 7:**
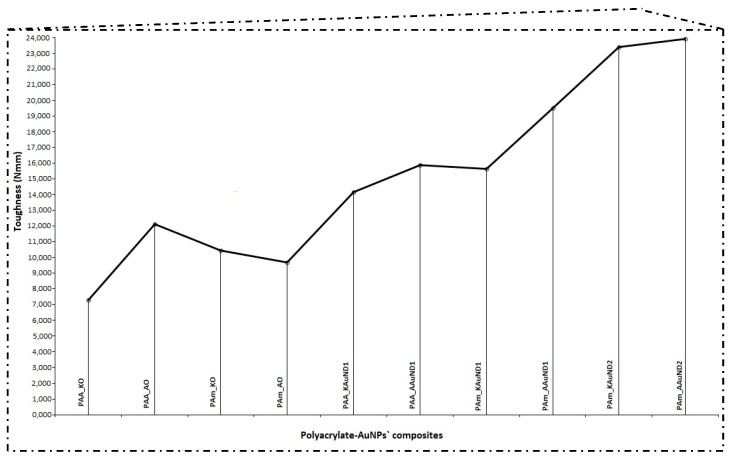
Mechanical properties (toughness) of control samples and polyacrylate-AuNPs’ composites.

**Figure 8:**
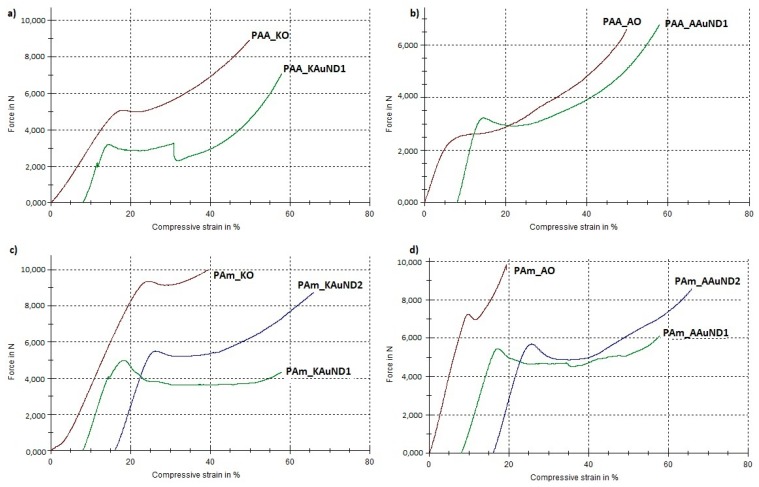
Load-compressive strain curves for tested specimens: (**a**) PAA_KO and PAA_KAuND1; (**b**) PAA_AO and PAA_AAuND1; (**c**) PAm_KO, PAm_KAuND1 and PAM_KAuND2; (**d**) PAm_AO, PAm_AAuND1 and PAm_AAuND2.

The compressive tests results of polyacrylate-AuNPs’ composites with the measured parameters (maximum load, compressive strength and toughness) are presented in Table 4, and graphically in Figure 7 and Figure 8. In general, the compressive strength behaviour of polyacrylate-AuNPs’ composites exhibited lower compressive strength values, while their toughness was substantially improved in comparison with the control samples. 

The compressive strength for composite PAA_KAuND1 (164.15 MPa) was an 18.9% decrease compared with the control sample PAA_KO (202.43 MPa), while the toughness of the composite (14,157 Nmm) increased by 94.0%, compared to the control sample PAA_KO (7292 Nmm). For composite PAA_AAuND1, compressive strength (161.76 MPa) decreased by 15.4% and toughness (15,877 Nmm) increased by 31.1% as compared to the control sample PAA_AO (191.30 MPa and 12,108 Nmm). On the other hand, for the composite PAm_KAuND1, the compressive strength (104.22 MPa) decreased by 43.8%, while its toughness (15,647 Nmm) increased by 49.5%, compared to the control sample PAm_KO (185.50 MPa and 10,464 Nmm). Similar results were observed for composite PAm_AAuND1, where the compressive strength (115.74 MPa) decreased by 35.9%, while toughness increased by 101.4% (19,517 Nmm) compared with the control sample PAm_AO (180.44 MPa and 9689 Nmm). The compressive strength for composite PAm_KAuND2 (165.21 MPa) decreased by 10.9% and the toughness of composite (23,386 Nmm) was increased by 123.5% as compared with the control sample PAm_KO (185.50 MPa and 10,464 Nmm). Meanwhile, the decrease in compressive strength for composite PAm_AAuND2 (179.28 MPa) was not large, as the compressive strength decreased only by 0.64% compared with the control sample PAm_AO (180.44 MPa). The toughness of this composite was also the highest (23,883 Nmm), increasing by 146.5% compared to the control sample PAm_AO (9689 Nmm).

Compressive tests revealed that, after 50% compression deformation, some of the samples fracture in ductile mode. Reduction of compressive strength and fracture of polyacrylate-AuNPs’ composites suggests that the incorporation of colloidal AuNPs into the polymer matrix decreased the strength of the material, while their toughness was substantially increased. This significant increase in polyacrylate-AuNPs’ composite was higher in polyacrylate-AuNPs’ composites containing AuAc AuNPs, and this increase can be attributed to the fact that AuAc AuNPs remained interconnected in the mesh even after being incorporated in the polymer matrix (no breaking of the bonds occurred), thus, it can be assumed that these AuNPs work as reinforcements, organised into a complex architecture, thereby strengthening the polymer matrix, resulting in improved mechanical properties [68]. On the other hand, AuNPs from AuCl were more dispersed (no mesh was observed), resulting in lower material toughness. The increased toughness implies that the incorporation of colloidal AuNPs greatly improved energy absorption for this material system, which is a major feature of polyacrylate-AuNPs’ composites for their use in dental applications. As can be seen from Figure 5c and Figure 6c, the polymer matrix already contains stochastically distributed air bubbles. The substantial enhancement in toughness for this material system could be that the air voids formed during the polymerization process, and the AuNPs, can slow down, or even prevent, a cracking process, because the micro crack tip stops at the surface of the air bubble or of the AuNP. For this material system, the air bubbles and AuNPs, (or even clusters of them), may serve as a crack blunter, which can lead to reduced brittleness and increased toughness of the composite, respectively.

Meanwhile, the compressive strength decrease is most likely attributed to the aggregation phenomena of AuNPs, and to the formation of air bubbles in the polymer matrix during polymerization. 

## 4. Conclusions

In summary, we reported the successful synthesis of colloidal AuNPs from two different gold metal salt precursors, gold (III) chloride and gold (III) acetate, respectively, using modified USP and their use in the preparation of polyacrylate-AuNPs’ composites. The synthesis of AuNPs was carried out under slightly different conditions and the effect of these parameters was characterised on the AuNPs’ morphology. The characterisation of colloidal AuNPs revealed that the AuNPs from AuAc were more ellipsoidal as compared to the spherical nature of AuNPs from AuCl, due to the collision and sintering of AuAc AuNPs during AuNPs’ synthesis. The prepared AuNPs were of different sizes and displayed a single absorption peak in the visible range, confirming their stability. 

Polyacrylate-AuNPs’ composites were prepared successfully by the free radical polymerization, initiated by two different radical initiators, APS and KPS, respectively. SEM characterisation showed that the AuNPs were not ideal but were relatively uniformly distributed through the polymer matrix and remained stable during polymerization. The density measurements revealed that the addition of colloidal AuNPs into the polymer matrix increased the densities of polyacrylate-AuNPs’ composites. On the other hand, the incorporation of AuNPs in the polymer matrix decreased their compressive strength, while their toughness increased significantly. This increase in composites’ toughness is due to the fact that AuNPs work as reinforcements, since they fill the spaces in the polymer chain adequately, thereby strengthening the polymer matrix. Increased composite toughness is a major feature of the prepared polyacrylate-AuNPs’ composites, since it limits their brittle fracture. Based on the obtained results, and due to the anti-inflammatory and antimicrobial properties of AuNPs, polyacrylate-AuNPs’ composites have potential for use in dentistry, such as temporary cavity fillings, since they can help with the treatment and prevention of various dental diseases, like toothache and root canal inflammations.

## Figures and Tables

**Table 1:** Parameters used in Ultrasonic Spray Pyrolysis (USP) synthesis.

Precursor ^a)^	Au Solution Concentration (g/L)	T_1_^b)^ (°C)	T_2_^c)^ (°C)	T_3_^c)^ (°C)	T_4_^c)^ (°C)	Gas Flow Rate (L/min)		Collecting Medium
						N_2_	H_2_	
AuAc_1	1	120	300	300	300	6	3	D.I. water
AuAc_2	1	120	350	350	350	5	2	D.I. water
AuCl_1	1	120	350	350	350	6	3	D.I. water
AuCl_2	1	120	400	400	400	5	2	D.I. water

^a)^ Precursor solutions: gold acetate (AuAc) and gold chloride (AuCl); ^b)^ T_1_: evaporation zone temperature; ^c)^ T_2_, T_3_, T_4_: reaction zone temperatures.

**Table 2:** Composition data for prepared control samples and polyacrylate-AuNPs’ composites.

Sample Name ^(a)^	Used Monomer	Monomer Mass (g)	m_NaOH_ (g)	V_H2O_ (mL)	Used AuNPs	Volume (mL)	Initiator	Mass (g)	Reducing Agent
PAA_KO	AA	2.63	1.18	5	/	/	KPS	0.05	TEMED
PAA_AO	AA	2.63	1.19	5	/	/	APS	0.05	TEMED
PAm_KO	Am	5.01	/	/	/	/	KPS	0.05	TEMED
PAm_AO	Am	5.00	/	/	/	/	APS	0.05	TEMED
PAA_KAuND1	AA	2.63	1.18	1.0	AuCl_1	4.0	KPS	0.05	TEMED
PAA_AAuND1	AA	2.63	1.20	1.5	AuCl_2	3.5	APS	0.05	TEMED
PAA_KAuND2	AA	2.62	1.15	1.5	AuAc_1	3.5	KPS	0.05	TEMED
PAA_AAuND2	AA	2.64	1.18	1.5	AuAc_1	3.5	APS	0.05	TEMED
PAm_KAuND1	Am	5.03	/	/	AuCl_2	5.0	KPS	0.05	TEMED
PAm_AAuND1	Am	5.02	/	/	AuCl_2	5.0	APS	0.05	TEMED
PAm_KAuND2	Am	5.00	/	/	AuAc_2	5.0	KPS	0.05	TEMED
PAm_AAuND2	Am	5.00	/	/	AuAc_2	5.0	APS	0.05	TEMED

^(a)^ Sample name: P**AA**_*K*O: Control sample prepared from acrylic acid (**AA**), with initiator potassium persulfate (*K*PS); P**AA**_*A*O: Control sample prepared from **AA**, with initiator ammonium persulfate (*A*PS); P**Am**_*K*O: Control sample prepared from acrylamide (**Am**), with initiator *K*PS; P**Am**_*A*O: Control sample prepared from **Am**, with initiator *A*PS; P**AA**_*K*AuND1: Sample prepared from **AA**, with initiator *K*PS, 1: Sequential number; P**AA**_*A*AuND1: Prepared from **AA**, with initiator *A*PS, 1: Sequential number; P**Am**_*K*AuND1: Sample prepared from **Am**, with initiator *K*PS, 1: Sequential number; P**AA**_*A*AuND1: Sample prepared from **Am**, with initiator *A*PS, 1: Sequential number.

**Table 3:** Densities of prepared control samples and polyacrylate-AuNPs’ composites.

Sample	Density (g/cm^3^)	Increase of Density, Compared to the Control Sample (%)
PAA_KO	1.3850	/
PAA_AO	1.2383	/
PAm_KO	1.5271	/
PAm_AO	1.4927	/
PAA_KAuND1	1.6234	17.2
PAA_AAuND1	1.2757	3.02
PAA_KAuND2	1.9482	40.6
PAA_AAuND2	1.6548	33.6
PAm_KAuND1	2.1710	42.1
PAm_AAuND1	1.5977	7.03
PAm_KAuND2	1.5666	2.58
PAm_AAuND2	1.6001	7.19

**Table 4:** Mechanical properties of control samples and polyacrylate-AuNPs’ composites.

Nr.	Sample	F_max_ (N)	Compressive Strength (σM) (MPa)	Toughness (Nmm)
1	PAA_KO	8105.15	202.43	7292
2	PAA_AO	6622.51	191.30	12,108
3	PAm_KO	10,012.40	185.50	10,464
4	PAm_AO	9833.40	180.44	9689
5	PAA_KAuND1	7078.82	164.15	14,157
6	PAA_AAuND1	6781.60	161.76	15,877
7	PAm_KAuND1	4980.09	104.22	15,647
8	PAm_AAuND1	6120.77	115.74	19,517
9	PAm_KAuND2	8750.12	165.21	23,386
10	PAm_AAuND2	8572.20	179.28	23,883
